# Walking together: women with the severe symptoms of menopause propose a platform for a walking program; outcome from focus groups

**DOI:** 10.1186/s12905-020-01037-y

**Published:** 2020-08-05

**Authors:** Beate C. Sydora, Tatjana Alvadj, Alexandra Malley, Maria Mayan, Tami Shandro, Sue Ross

**Affiliations:** 1 Department of Obstetrics and Gynecology, Faculty of Medicine and Dentistry, and Women and Children’s Health Research Institute, University of Alberta, Edmonton, T6G 2R3 Canada; 2 Department of Obstetrics and Gynecology, University of Alberta, 626-1 Community Service Centre, Royal Alexandra Hospital, 10240 Kingsway Ave, Edmonton, T5H-3V9 Canada; 3 Women and Children’s Health Research Institute, Faculty of Extension, University of Alberta, Edmonton, T6G 2R3 Canada; 4 Faculty of Extension, University of Alberta, Edmonton, T6G 2R3 Canada; 5 Family Medicine, Faculty of Medicine & Dentistry, Women and Children’s Health Research Institute, University of Alberta, Edmonton, T6G 2R3 Canada

**Keywords:** Menopause, Menopause symptoms, Walking, Walking program, Walking application

## Abstract

**Background:**

Menopause and midlife are stages in a woman’s life that can be marked by debilitating symptoms and increasing risks for cancer, cardiovascular, metabolic, and bone health issues. Walking represents a simple, low cost, and widely accessible activity with proven health benefits, though its therapeutic effect on alleviating menopause symptoms is not well characterized. Women are generally not opposed to exercise programs; however, increasing or maintaining exercise levels remains a challenge. We undertook a qualitative descriptive study to explore features of a walking program that would be conductive to menopausal women’s participation, as well as to inform the development of such a program.

**Methods:**

We conducted focus groups with women recruited from two menopause clinics and who suffered from moderate to severe menopause symptoms. The focus groups were audio recorded and transcribed. Women were prompted to talk about their menopause experience and exercise practice and how they would envision a walking exercise program that would keep them engaged. Qualitative content analysis was used to analyze the data and to identify characteristics of a walking exercise program.

**Results:**

Twenty women participated in 5 focus groups. Women were very interested in trying walking as a means of staying healthy and possibly reducing menopause symptoms. Four major characteristics emerged as important for a walking program: (a) sensitivity to health realities of menopausal women, (b) inclusivity of various needs/levels of physical ability, (c) attentiveness to the need for mutual social support, (d) flexibility in planning of locations and scheduling.

A restricted social network platform with features catering to women in menopause was suggested as suitable to initiate and sustain an adequate walking program.

**Conclusions:**

The findings of this study will be essential in designing a program that would be attractive for women to start and maintain a walking habit. The program would assist in elucidating whether walking is a useful and valuable alternative therapy for menopausal symptoms and, ultimately, might help women staying fit in midlife and postmenopausal.

## Background

Menopause is a natural stage in a woman’s life, characterized by the cessation of menstruation and the end of the reproductive years. Hormonal changes during the menopause transition period are often accompanied by physical and psychological symptoms that can impact women’s quality of life (QOL) [1]. During midlife, women also tend to reduce their exercise rates, accompanied by a reduction in basal metabolic rate and loss of lean muscle. This increases their risk for weight gain and obesity, which is associated with comorbidities including diabetes, hypertension, cardiovascular disease, and cancer [2–4].

Walking represents a widely accessible, low cost approach to exercise with proven health benefits [2, 5]. Walking has the potential to be incorporated into most people’s lives [6]. The low risk of injury associated with walking can allow individuals to remain active into older age [7–9].

Research indicates that women are not averse to regular exercise, especially those seeking ways to avoid the perceived increased health risk of hormonal therapy [10, 11]. Nevertheless, increasing women’s exercise levels remains a challenge [12, 13]. Common barriers to initiating or increasing exercise include competing time demands, safety concerns, weather, and not having an exercise partner [14, 15]. Walking is the preferred type of exercise among menopausal women [16].

Designing a walking program that is attractive and engaging for women in menopause transition or postmenopausal can be challenging. A variety of components must be considered, including walking group dynamics, program frequency, time, location, and environmental and climate challenges. A city’s layout and general walkability can be an important factor in inspiring or deterring people from walking [17, 18].

The Mature Women’s Health Research group at the University of Alberta aims to investigate whether a walking exercise program can improve QOL in women with moderate to severe menopause symptoms who are seeking help at Edmonton’s menopause clinics. Before exploring the characteristics of an effective walking exercise program for women, we carried out a scoping review of relevant literature and undertook an exploratory environmental scan of existing Edmonton walking programs.

Our scoping review confirmed that participating in regular walking programs can have beneficial effects on menopause symptoms and risk factors arising around the age of menopause transition. Our review was unable to establish minimum session length and walking intensity, but we did find that exercise persistence and adherence were important to experience benefit [19].

Our environmental scan of local walking resources identified a variety of groups and organizations that engage in regular, year-round walking exercise, but none was specifically designed for women in the age of menopause transition [20].

Focus group studies have been successfully employed to gain insight into individual experiences and perceptions of exercise programs and to provide information for the development of programs accordingly [21, 22]. Using a similar approach to inform the design of a walking program, we conducted a qualitative study with the research question: What key characteristics need to be included in planning and developing a walking program specific for women with moderate to severe menopause symptoms?

## Methods

### Study design

We used a qualitative descriptive design which enables researchers to investigate, describe and summarize a phenomenon in everyday terms [23]. Focus groups were appropriate for this study to provide participants with the opportunity to interact with each other, to explore the topic at hand from a variety of viewpoints [24] and to “co-construct the meaning” of the walking program for women in menopause through “collective sense-making” [25].

### Recruitment

Women eligible to join the focus groups were those in menopause transition or postmenopausal, who attended one of the two Edmonton Menopause clinics, and were physically able to walk without assistance. Focus groups were formed using a purposeful sampling approach that identifies information-rich participants [26]. We were interested to learn from women who experienced moderate to severe menopause symptoms, who self-reported a wide range of current physical activity, from being completely inactive to being very active and who were interested in the topic of the study. Women were approached by clinic staff between July and October 2017: those interested to take part were invited to join scheduled focus groups. Written consent was collected from all women at the time of the focus groups. The study was approved by the University of Alberta Health Research Ethics Board (Pro00072912).

### Data collection

Two focus group moderators were present. One, with training in moderating focus groups, and no prior relationship with the clinical or research team members, facilitated the discussions. The other was a research team member, who took notes on non-verbal data. A semi-structured discussion interview guide was used including open-ended questions on (i) attitudes towards exercise and walking, (ii) exercise barriers, (iii) exercise promotors, and (iv) perceptions about an ideal walking program. The focus groups were audio recorded and transcribed verbatim. In the process of transcription, all identifying information was replaced by codes, such as P1, FG1 (Participant 1, Focus group 1). Focus group notes that included non-verbal data, were taken to support the analysis.

### Data analysis

Qualitative content analysis, typically used in qualitative descriptive studies, was the approach used to analyze the focus group data [23]. Qualitative content analysis is oriented toward summarizing the information contained in the transcripts [27] through the systematic process of coding and identifying patterns [28].

The focus groups were analyzed iteratively and simultaneously with data collection. Analytical attention was primarily on the interaction between the participants and the inter-group dynamics [24]. At the same time, and given the composition of the groups (a mix of self-reported active and less active or inactive women), attention was given to the distinct individual voices and intra-group differences [24]. Open coding and identifying preliminary categories of data within individual focus groups were followed by the subsequent comparison of these initial categories across the focus groups. This preliminary analysis, conducted by the team member trained in qualitative data analysis, was discussed in a series of research team meetings: the emerging categories were checked for internal and external consistency and four key characteristics relevant to designing a walking program for women with moderate to severe symptoms of menopause were identified. The findings were presented to the participating women during the special session organized to validate of study results and further plan the program. In addition to practicing iterative data collection and analysis [29] as well as by regular peer/team interpretation, rigor was determined by maintaining methodological coherence, ensuring appropriate and sufficient sampling, and an audit trail [30]. These strategies ensured that the findings were logical and accurate representations of the data.

## Results

Twenty women participated in five focus groups held during December 2017 to February 2018 in two health care locations in Edmonton. Groups ranged in size from two to six women.

### Participants’ characteristics

Characteristics of the participants are presented in Table 1. The majority of women were aged in their 50s, had attended higher education, worked full time and were married or living with a partner. The women were in menopause transition or post-menopausal. The group reported a wide range of physical activity, from high to low (including 2 self-proclaimed “*couch potatoes*”).

**Table 1:** Demographics of participants from five focus groups

Participants characteristics	Participants *N* = 20
Age at time of focus group, [years (mean ± SD)]	52.4 ± 6.3
Level of education [N (%)]	
High School	5 [25]
College or University	15 [75]
Employment status [N (%)]	
Work full time	13 [65]
Work part time	4 [20]
Not in paid work	3 [15]
Marital status [N (%)]	
Married/living with partner	12 [60]
Single, living with others	5 [25]
Single, living alone	3 [15]
Menopause status [N (%)	
Still menstruating	2 [10]
Stopped menstruating	18 [90]
Self-reported level of physical activity [N (%)]	
Low ^a^	9 [45]
Moderate ^b^	6 [30]
High ^c^	5 [25]

^a^ no formal activities, housework, gardening, occasional walking

^b^ regular walking plus occasional other fitness such as swimming

^c^ regular fitness programs (yoga, barre, treadmill) several times per week

All participants suffered from severe, often multiple, symptoms of menopause that had significantly impacted their QOL over an extended period of time. Insomnia, night sweats/hot flashes and fatigue were the most prevalent symptoms discussed. In addition, lack of sex drive and vaginal dryness, memory loss, depression, and lack of motivation were described as troubling symptoms. Participants stated that the severity of the symptoms and their impact on the QOL were undermined by their primary care physicians. Most participants also reported feeling socially isolated, without connection to women with similar menopause problems.

In this context, participants discussed the features of a walking program that would be suitable for women in menopause. Four distinct characteristics emerged: (A) sensitivity to health related realities of women in menopause; (B) inclusivity of various expectations and levels of readiness (fitness) among participating women; (C) attentiveness to the need for social support, and (D) flexibility in planning locations and scheduling of the program.

### (A) Sensitivity to the health realities of women in menopause

The current health status and ability to be physically active varied among participating women. Participants who reported being able to exercise, described a positive impact on their wellbeing, such as improved sleep and better mental stability. Others, who were more active in the past, but developed co-morbidities or injuries, felt regret that they had to reduce the intensity and/or type of exercise. Participants who reported currently being less active, highlighted symptoms of fatigue and lack of energy as the de-motivating factor to exercise, stating “*Tired women will not walk”* (P2, FG5) and *“You’re too tired to do it”* (P1, FG5).

Walking was perceived by the participants across all five focus groups as a type of exercise that could be easy, accessible, relaxing, less risky for the body, and “*open to any level*” (P1, FG5). Still, many worried that the incapacitating nature of their menopause symptoms described by one participant as “*struggling to get out of bed in the morning*” (P4, FG2) as well as other health issues would be a potential barrier to joining a walking program. Therefore, the participants suggested that the walking program should be sensitive to different health realities among menopausal women, such as fatigue, “*the worry about injury”* (P1, FG3) (especially during the winter), and possible overheating/hot flushes while walking.

“ *I get so hot and then I get really irritated when you get that hot and you're irritated and when you can only take off so much clothes, right”* (P2, FG1).

Several suggestions were put forward to overcome these challenges such as indoor walking as a safer winter solution, cooling aids to manage overheating and hot flashes and basic coaching to prevent injury.

*“…but I’m thinking for people who have not been active, the worry about injury and just having somebody who can maybe coach them through that a little bit and what to expect or how to stretch out or what to watch for because even though we talk about walking and it seems like a simple thing, I wonder if it’s possible to have walking injuries”* (P1, FG3).

In addition, as some of the participants stated, the program should incorporate a right kind of encouragement to keep women motivated, included, and overcoming their concerns.

### (B) Inclusivity of various expectations and levels of fitness

In the context of these health realities, the focus groups further discussed the program’s goals and expectations. While there was a range of opinions how specific the goals of the walking program should be, the participants believed that the sense of clear expectations and purpose should be transparent, “*[…] something that keeps us in check as to what the purpose of the walk is”* (P6, FG2).

Some participants stressed the need for disciplined commitment to the program’s schedule, emphasizing the importance of predetermined health outcomes, “*something to achieve, you know, to look forward to*” (P2, FG2) to ensure that the program will “*make a difference*” (P3, FG1).

*“I also think being out and getting out giving you a sense of accomplishment that you know that I'm you know I'm doing this for me and I'm committed to it and I'm actually getting it done. I want some real rewards…”* (P4, FG2).

A more flexible approach was suggested by others. Some participants believed that establishing high-achieving goals may be attractive to some women, but these could also deter less active and less competitive women. The program therefore should respect women’s different initial fitness, offering different levels of walking intensity and leave room for individual goals.

“*As somebody who like I said is just starting to be a functioning human being again […] the expectations [should not be] too great […]so that they can start out small but grow as it can like expand as they feel like they can take on more”* (P4, FG2).

“*I like to be able to keep track of my steps, that’s my own personal goal so I think if people had individual goals that’s great too”* (P1, FG3).

While developing specific goals would be important for some participants, others would be comfortable with a more general purpose of *wellness*, envisioning a less competitive program that will “*improve health*” (P2, FG4) or contribute to “*feeling better*” (P1, FG5). Openness to and inclusion of women with various expectations and levels of fitness surfaced as consequential characteristics of the walking program.

### (C) Attentiveness to the need for social support

In discussion about the appropriate nature of the program, the importance of the social support to women, described by one participant as connecting with someone who “*is walking the same steps as I am*” (P2, FG5), was emphasized across all focus groups.

The participants envisioned an organized, regularly scheduled walking group that would be fun, with an optional “*coffee time*” at the end. Such a program would simultaneously provide an opportunity for physical activity and mutual emotional and social support to women who often do not have a place to share their menopause experience. This was discussed for example in FG3:

“*[…] and it’s not necessarily about the walk […] it’s about the socialization*. (P3) “*Yeah that’s a big part*” (P4). “*Because that helps, it’s not the exercise that helps, it’s the socialization and realizing that they're not alone”* (P3). “*[…] I think if the main focus is socialization, just all in the same boat, but we’re going to walk down the block and talk about our menopausal symptoms, […] the walking is the secondary piece”* (P2).

The concept of a “*buddy system”* was also broadly discussed across the focus groups in the context of the lack of motivation to be physically active that women often experience. One exchange, which occurred in FG1, suggested that peer encouragement and mutually developed accountability to one another would be an incentive to continued participation:

“*I would need a buddy because I wouldn't want to do it on my own*” (P3). “*Walking partners are great”* (P1). “*That’s true too*” (P2). “*I would need a buddy. I think definitely the, you know like, group or buddy I think it would, like for me that would be great*. *[…]and yeah to be accountable to go, yeah there’s an accountability piece, there’s a social piece, there is just that… Yeah there’s a lot of features to the buddy thing”* (P3).

An additional suggestion, made in two focus groups described a virtual community that would use a Facebook and/or a mobile application accessible to all its members. This was described as multipurpose tools that could increase motivation, keep the social network engaged, and keep track of individual achievements.

*“And if you set it up something like you know on one of the social aspects on Facebook whenever you set a group up and say ok this is the plan for the day, this is where everybody is going, love to see you there, you know and kind of just whoever shows, shows. You know it’s not a hardcore that you have to be there, it’s you know feel free when you feel up to it or when you're having a day or when you just need to talk”* (P3, FG3).

While most participants anticipated a face-to-face walking program that provides social group activity, some participants mentioned the possibility for a combination of group and individual activities. In FG4, this was suggested as an additional opportunity to “*clock our own walking*” (P2, FG4). In FG2, two participants (one who lives out of town, and one self-described “*loner*”), proposed a virtual community where participants would exercise on their own, but could connect periodically in person with the rest of the “walkers”:

*“…they should make a menopause walking app or something so you check in or something like that”* (P3, referring to MyFitnessPal). “…*if we did something like that and there was an app that could be adapted to be used then you're doing it alone but you're not alone”* (P2). “*I would want to see how everyone else is doing you know like P1 did 10,000 steps or she walked 10 miles this week. […] so if there’s an app […] and if we’re all willing to provide that information […] to motivate us* (P6).

By developing in- person and virtual community, as well as individual and group activities, the walking program would demonstrate adaptability and flexibility to suit different lifestyles, while simultaneously creating a space for social interaction.

### (D) Flexibility in planning locations and scheduling

The weather and seasonal changes were emphasized as a key aspect of developing a walking program. The severity of winters with low temperatures and icy sidewalks on one hand, and hot summer days on the other, call for flexibility in identifying appropriate locations and venues for walking. All focus groups discussed summertime walking along the many city walking trails, except when the temperature is too high and walking would be more comfortable in air-conditioned spaces.

During the winter, walking inside was preferred as a safer option by the majority of participants, for example walking in recreation centers or shopping malls; though some participants felt inside walking was a less attractive option.

Discussions about geographical locations for the walking program provided less consistent suggestions. While some of the participants were willing to drive and meet the group wherever it is located, the majority would prefer the program being closer to home, to save time and avoid unnecessary driving. The following exchange in FG4 exemplifies this point:

“*Well I guess it depends on where the women are all located right? You want to make it feasible, like you want to make it, you know, easily accessible; if somebody has to travel a half an hour plus to get there, it’s best to sort of have it in three to four different areas in the city”* (P2). “*I totally agree with that […] because if there was a walking group that just met downtown I wouldn’t join it because I live [*in the part of the city*] that’s a half an hour drive to get there”* (P1). *“I agree, I wouldn't drive far to go for a walk because I would just go for a walk out of my house”* (P5). “*I’m a driver so that wouldn't bother me; that would be fine for me”* (P2). “*It’s just the time I think for myself anyways you know it’s just, it’s making the time”* (P4).

Possible solutions included providing the program simultaneously in different neighborhoods or parts of the city, or be mobile and move around the city on a weekly basis, with the various time schedule options, such as evening walking (during the week) and mid-days or afternoons (on weekends).

As participants discussed a number of factors related to the logistics of the planning (weather, location, time), the need for accommodation and flexibility has emerged across all focus groups, to ensure the access and commitment to and feasibility and sustainability of the program. Most important for all women was the commonality of menopause for the walking group; as one woman sums it up: “*There is always the one constant, it’s the menopause group, so that constant there”* (P1, FG1).

## Discussion

The information collected in this focus group study are crucial for the development and implementation of a sustainable walking program that could be therapeutic for women suffering from menopause or menopause-related symptoms. Women were eager to participate and discuss a program that would serve their needs. We heard that women with severe symptoms of menopause often feel they experience insufficient social support. Any planned walking program should acknowledge the need for women to share their experiences and the strategies they use to cope with menopause.

The women in this study reiterated findings from previous research, suggesting that women are keen to use walking as a form of exercise that is easy, safe and affordable and an accessible way to be physically active [5, 7]. They also considered many characteristics of a walking program that would be important for menopausal women. These considerations included taking into account different incapacitating menopause or other symptoms and varying levels of fitness. Programs should provide walks of different distances and degrees of efforts to cater for women unused to walking or who were less prepared for exercise, as well as more active women. They suggested including clear program objectives and goals to help women to gradually build strength and fitness.

Many of the features highlighted in the focus groups are already addressed by the existing local walking programs, including mutual support, regular and varied walks [20]. However our focus group participants believed that a program for menopause-specific groups was needed for women to share their menopause experiences and to provide mutual self-support that is safe, motivating, and creative in solving the problems specific to women in menopause.

To achieve the proposed program features, several possibilities exist from face to face contact, telephone scheduling, or – as suggested in two of the focus groups - through Facebook or other social network applications that could be specifically set up. While a study by Daley claims that use of technology to deliver exercise interventions is not popular among menopausal women [16], a more recent survey found that 7 in 10 Canadians over the age of 65 feel confident about technology use and 86% are online daily [31]. All the women in the focus group were comfortable with the use of smart phones for scheduling and social interactions.

One interesting and novel solution suggested by focus group participants, was the development of a virtual community-based online app that would be restricted to women who join the walking groups. The app would permit women to choose their own degree of involvement based on their own needs or preferences for communal walking and/or individual walking. The element of social support and accountability would be important. Women would be able to link up with others who lived in the same neighborhoods and a buddy system could become a feature incorporated in the app to address these points.

A key consideration in designing a walking program was flexibility in logistics and adaptability to the weather and daylight changes; the latter being a major concern in a Northern city such as Edmonton. Attention to elements of the environment such as natural surroundings and neighborhood walkability and convenience have been shown to increase physical activity levels [32]. An app could be linked to local maps and events and include reports on weather condition.

Our research group has focused further efforts to create a walking program for women in menopause, by collaborating with computing scientists to develop an online application that will satisfy the preferences identified during the focus groups. To be consistent with our main focus of creating a therapeutic program for menopause symptoms, the application will include links to proper menopause resources and self-assessment tools. A research component of the application will allow menopause researchers to enroll and follow-up with consented women via surveys and questionnaires. A prototypic application has been developed and is currently being tested.

### Study limitation

The lack of diversity among participants is the most noticeable limitation of this study. The women, recruited from either of the two Edmonton Menopause clinics, typically suffer from moderate to severe menopause symptoms in addition to a variety of other health problems [33]. Even though within our apparently homogeneous sample we did identify a range of needs and logistical difficulties that would be important for others to consider in creating a walking program. We believe that future research needs to put more effort on including women who may enhance or challenge our established understanding of the experience of menopause and exercise. We also acknowledge the barriers that were imposed by the severe weather in the location where the study was conducted, which in some cases limited the anticipated number of focus groups participants.

### Study strength

Research has shown that interventions to promote walking are successful in increasing walking as a physical activity [9]. A strength of the study is the close analytical attention to participants’ individual perspectives, as well as interpersonal interactions during the focus groups and intra-group differences, which contributes an understanding of the complexity of the issue and helps to identify barriers and enablers for exercise [15]. Our findings provide ideas for the development of walking programs and inform us about general features that women in menopausal stages would find both attractive and feasible.

While originally intended to inform the development of a program in Edmonton, Alberta, it is likely that these factors would also provide a useful starting point for others designing similar programs.

## Conclusion

The findings of our study have highlighted characteristics that menopausal and postmenopausal women find important for a walking program. The results will assist in our goal of designing a program in the Edmonton area that would provide year-round walking opportunities and would be engaging and inspiring to menopausal and postmenopausal women to start and maintain a walking habit. The program would assist in elucidating whether walking is a useful and valuable alternative therapy for menopausal symptoms. This program ultimately has the potential for being widely available in a range of climates and physical locations, and of generating a meaningful and sustainable impact on health outcomes for women in menopause transition and postmenopausal in encouraging healthy behavior and preventive physical activity.

## Data Availability

The data pertaining to the current study are available from the corresponding authors in accordance with appropriate data use agreements and IRB approvals for secondary analyses.

## Abbreviations

QOL: Quality of life
P #: Participant #
FG #: Focus group #
